# Supplementary material for: Hemocyanins: Microscopic Giants with Unique Structural Features for Applications in Biomedicine
Source: Vaccines (Basel). 2025 Oct 23;13(11):1086. doi: 10.3390/vaccines13111086 (PMC12656151; doi:10.3390/vaccines13111086)
Supplement: Supplementary file 1 [file vaccines-13-01086-s001.zip › vaccines-3904962-supplementary.pdf]

Supplementary Table S1. **Overview of hemocyanins from various mollusk species.**

The table provides an overview of hemocyanins from various mollusk species. It includes details on the source, structural characteristics, molecular mass, immunological and biological effects, applications, and key references. Structural features encompass subunit composition, oligomerization state, thermal stability, and glycosylation profile when available. Molecular mass is reported for both oligomers and individual subunits. The biological effects include immunostimulatory, antitumor, and antimicrobial properties, as well as vaccine carrier functions, observed in preclinical and clinical studies, and in cell lines. Applications highlight the use of these hemocyanins as immunogens, carriers for hapten and peptide conjugates, adjuvants, and model glycoproteins for investigating glycan-dependent immune mechanisms. The references correspond to studies that support these findings.

Hemocyanin acronym	Source	Structure	Molecular mass	Reported effects	Applications	References
KLH	*Megathura crenulata*	- Homodidecameric structures: KLH1 and KLH2.<br>- High thermal stability (Tm of 67°C).<br>- Glycan content: 3.4% (w/w) for KLH2, with less mannose and more N-acetylgalactosamine than KLH1, whose glycan content comprises 3.0 % (w/w).	- Didecamer: ~8 MDa.<br>- Subunits: KLH1 ~390 kDa, KLH2 ~350 kDa.	- In clinical trials, KLH elicited specific T and B-cell responses, acting as an immunogenic neo-antigen in cancer vaccines.<br>- KLH showed anti-proliferative effects in cell lines of breast, pancreatic, and esophageal cancer.<br>- In animals with an ascites tumor of Guerin, KLH increased splenic lymphocytes and prolonged survival time.<br>- Conjugation of small molecules to KLH enhanced immunogenicity, facilitating the generation of high-titer antibodies. These effects have been partially explained due to KLH binding to TLR4, MR, and DC-SIGN in a glycosylation-dependent manner.	- Nonspecific immunostimulant for superficial bladder cancer; currently being tested in clinical trials of therapeutic cancer vaccines for humans, including those targeting melanoma, breast cancer, and bladder carcinoma.<br>- Carrier protein for research and development of antibodies against haptens, such as dopamine, DNP, β-amyloid, and GnRH peptides.<br>- Model glycoprotein for the study of antigen processing, dendritic cell activation, and glycan-dependent immune mechanisms.	- Lamm et al. J Urol. 1993.<br>- Swerdlow et al. J Urol. 1994.<br>- Swerdlow et al. Comp Biochem Physiol B Biochem Mol Biol. 1996.<br>- Markl et al. J Cancer Res Clin Oncol. 2001.<br>- Idakieva et al. Comp Biochem Physiol B Biochem Mol Biol. 2004.<br>- Dolashka et al. Immunol Invest. 2011.<br>- Swaminathan et al. Br J Clin Pharmacol. 2014.<br>- Wimmers et al. Sci Rep. 2017.<br>- Saghari et al. Frontiers in Drug Discovery. 2022.<br>- Díaz-Dinamarca et al. Pharmaceutics. 2022.
CCH	*Concholepas concholepas*	- Heterodidecameric structure, comprised of two subunits: CCH-A and CCH-B.<br>- Stabilization does not require Ca(2+) or Mg(2+) in the medium.<br>- High thermal stability (Tm = 78.0°).<br>- Glycan content: 3.6% (w/w) for CCH-A, and 2.5 % (w/w) for CCHB; heterogeneous mannose-rich N- and O-glycosylations.	- Didecamer: ~8 MDa.<br>- Subunits: CCH-A: 405 kDa; under reducing conditions, it is cleaved to CCH-A1 (300 kDa) and CCH-A2 (108 kDa). CCH-B: 350 kDa.	- CCH binds MR, DC-SIGN, and TLR4 in a glycosylation-dependent manner, promoting endocytosis and cytokine secretion, upregulation of costimulatory molecules, and antigen presentation.<br>- CCH increased specific IgG and reduced tumor size in murine models of melanoma, oral cancer, and superficial bladder cancer. Furthermore,<br>- CCH promoted mRNA expression of MHC II, IL-12a, TNF, and IFN-γ1-1 in zebrafish, protecting against *F. noatunensis subsp. orientalis.*<br>-	- Adjuvant in TAPCells®, the Chilean dendritic cell vaccine against human melanoma and prostate cancer.<br>- Carrier in the formulation of veterinary immunocontraceptive vaccines.<br>- Carriers to the development of antibodies against peptides and haptens.<br>- Nonspecific immunostimulant for cancer research in animal models (melanoma, superficial bladder cancer, and oral cancer).<br>- Model glycoprotein for the study of antigen processing, dendritic cell activation, and glycan-dependent immune mechanisms.	- De Ioannes et al. J Biol Chem. 2004.<br>- Manosalva et al. Hybrid Hybridomics. 2004.<br>- Moltedo et al. J Urol. 2006.<br>- Becker et al. Intl Immunopharmacol 2009<br>- Nakamura et al. Biosensors (Basel). 2013.<br>- Salazar-Onfray et al. Biol Res. 2013.<br>- Lagos et al. Fish Shellfish Immunol. 2017.<br>- Palacios et al. Eur J Med Chem. 2018.<br>- Mora Romá, et al. J Immunol Res. 2019.<br>- Gleisner et al. J Immunother Cancer. 2020.<br>- Idakieva et al.. Biotechnology & Biotechnological Equipment 2020.<br>- Poirier et al. PLoS One. 2021.<br>- Muñoz SM, et al. Structure. 2024.
RvH	*Rapana venosa or Rapana thomasiana are the same species*	- Homodidecameric structures: RvH1 (reassociates in tubules) and RvH2 (reassociates into multidecamers, tubules, and didecamers).<br>- Glycan content: 8.9% (w/w); 12,8% for RvH1 and 4,4% for RvH2. N-	- Didecamer: ~8 MDa.<br>- Subunits RtH1 and RTH2	- RvH have apoptotic and antiproliferative effects in bladder cancer, colorectal carcinoma cell lines, and in the Graffi myeloid tumor model.<br>- Oxidized-hemocyanin induced tumor suppression in a murine melanoma model, increasing NK, CTL, and macrophage infiltration.	- Nonspecific immunostimulant for cancer research in cell lines and animal models (ascites tumor of Guerin and Graffi myeloid tumor model).<br>- Carrier for research and development of antibodies.<br>- Antimicrobial protein with potential antiviral effects in vitro.	- Dolashka-Angelova, et al. Biochim Biophys Acta. 2003.<br>- Dolashki et al. Biochim Biophys Acta. 2008.<br>- Dolashka-Angelova et al. Immunol Invest. 2008.<br>- Dolashka-Angelova et al. Bioconjug Chem. 2009.

		glycosylations are complex heterogeneous high-mannose glycans with unusual acidic terminal structures.		- RtH induced IgG, tumor-specific CTLs, and decreased tumor growth while prolonging life span in an animal model of colon carcinoma.<br>- RvH promoted specific antibodies against hemagglutinin from the influenza virus.<br>- Fractions of RvH had antimicrobial potential against live *E. coli* .<br>- The glycosylated FU RvH2-e showed antiviral activity against herpes simplex virus type 1, and the glycosylated FU RvH- against the respiratory syncytial virus.<br>- RtH2 induced late apoptosis/necrosis in cells infected by herpes simplex virus.	- Immunostimulant protein with potential radioprotective effects in animal models.	- Dolashka-Angelova et al. Bioconjug Chem. 2009.<br>- Antonov et al. J BUON. 2015.<br>- Georgieva et al. Biomedicines. 2020.<br>- Georgieva A, et al. Biomedicines. 2023.<br>- Kirilova et al. Pharmaceuticals (Basel). 2024.
FLH	*Fissurella latimarginata*	- Homodidecameric structure, comprised of only one subunit.<br>- Glycan content: >4% with heterogeneous mannose-rich N- and O-glycosylations.	- Didecamer: ~8 Mda.<br>- Subunit: 350 kDa.	- Similar to KLH and CCH, FLH binds MR, DC-SIGN, and TLR4 in a glycan-dependent manner, regulating the proinflammatory response and antigen presentation.<br>- FLH promotes specific antibody responses and exhibits potent antitumor effects in murine models of melanoma and oral cancer.<br>- FLH stimulated the proliferation of T cells from OT-I and OT-II murine models when administered with OVA.	- Nonspecific immunostimulant for cancer research in animal models (melanoma and oral cancer).<br>- Safe and effective adjuvant in animal models.<br>- Model glycoprotein for the study of antigen processing, dendritic cell activation, and glycan-dependent immune mechanisms.	- Arancibia et al. PLoS One. 2014 .<br>- Zhong et al. J Immunol. 2016.<br>- Palacios et al. Eur J Med Chem. 2018.<br>- Jiménez et al. Front Immunol. 2019 .<br>- Villar et al. Eur J Immunol. 2021.<br>- Díaz-Dinamarca et al. Pharmaceutics. 2022.
HlH	*Helix lucuorum*	- Homodidecameric structure is comprised by three isopolypeptides: beta-HlH, alpha(D)-HlH and alpha(N)-HlH.<br>- Glycan content: contains heterogeneous mono- and bi-antennary N-glycans, with and without core-fucosylation.<br>- High conformational stability (Tm = 82.5°C), within pH values of 6.5−8.0, and in the presence of Ca(2+) and Mg(2+).	- Didecamer: >9 MDa.<br>- Subunit: beta-HlH 1068 kDa. alpha(D)-HlH and alpha(N)-HlH: and 1079 kDa.	- HlH decreased the proliferation of cell lines from human bladder cancer, ovarian cancer, acute monocytic leukemia, prostate cancer, glioma cancer, colorectal carcinoma, and Burkitt's lymphoma.<br>- HlH downregulated metastatic genes and upregulated apoptotic genes in bladder cancer cells. Its conjugates induced cytotoxicity on breast cancer cell lines. It increased splenic lymphocytes and prolonged the survival time of tumor-bearing animals with the ascites tumor of Guerin. HlH promoted specific antibodies as well as antiproliferative and apoptogenic effects in the Graffin myeloid tumor model.<br>-	Nonspecific immunostimulant for cancer research in several cell lines and animal models (ascites tumor of Guerin and Graffi myeloid tumor model).	- Keller et al. Eur J Biochem. 1999.<br>- Velkova et al. Comp Biochem Physiol B Biochem Mol Biol. 2010.<br>- Dolashka P, et al. Immunol Invest. 2011.<br>- Kostadinova et al. J Fluoresc. 2013.<br>- Boyanova et al. Biomed Rep. 2013.<br>- Antonova et al. Z Naturforsch C J Biosci. 2014.<br>- Antonova O, et al. J BUON. 2015.<br>- Georgieva A, et al. Biomedicines. 2020.<br>- Dolashka P, et al. Biomolecules. 2020.<br>- Idakieva, et al. Biotechnology & Biotechnological Equipment 2021.<br>- Georgieva A, et al. Biomedicines. 2023.
HaH	*Helix aspersa*	- Didecameric structure. Two isoforms have been characterized: α-HaH (formed by two subunits αN-HaH and αD-HaH) and βc-HaH (formed by one subunit βc-HaH).<br>- High thermal stability (Tm ~80°C).	- Didecamer: ~8 MDa.<br>- Subunits have a similar Mr of ~450 kDa.	- Antimicrobial effect against *S. aureus*, *S. epidermidis*, and *E. coli*.<br>- Potent antineoplastic activity in cell lines of colorectal carcinoma.<br>- Oxidized-HaH induced tumor suppression in a murine melanoma model, increasing the infiltration of NK cells, melanoma-specific CTLs, IgM, and type 1 macrophages on tumors.	- Nonspecific immunostimulant for cancer research in cell lines and animal melanoma models.<br>- Carrier for ganglioside mimotope.<br>- Immunostimulant protein with potential antimicrobial effect.	- Todinova S, et al. J Anal Methods Chem. 2018.<br>- Georgieva A, et al. Biomedicines. 2020.<br>- Stoyanova et al. Mar Drugs. 2024

				- As a carrier of the ganglioside mimotope GD3P4 peptide, HaH decreased tumor growth and incidence, increasing infiltration of tumor-specific T cells in the spleen.		
HtH	*Haliotis tuberculata*	- Two isoforms have been identified: HtH1 and HtH2. For HtH1 the predominant oligomer is the didecamer, dependent on Ca(+2) and Mg(+2), while multidecamers were detected for HtH2.<br>- High thermal stability (Tm = 76.0°C).<br>- Glycan content: 4.5% (w/w).	- Didecamer: ~8 MDa.<br>- Subunits have a similar Mr of ~400 kDa.	Synthetic peptides based on HtH impaired the growth of *Bacillus subtilis* and *Erwinia carotovora*.	Hemocyanin with potential antimicrobial effect.	- Dolashka et al. Immunol Invest. 2011.<br>- Keller H, et al. Eur J Biochem. 1999.<br>- Harris et al. Micron. 2000;<br>- Zhuang et al. Dev Comp Immunol. 2015.<br>- Markl et al. J Cancer Res Clin Oncol. 2001.
HpH	*Helix pomatia*	- Didecameric structure. It consists of three components: two α-components (αD-HpH and αN-HpH) and a β-component (β-HpH).<br>- High thermostability (Tm = 80°C for functional units).<br>- Glycan content: 7% (w/w) for β-HpH.	- Didecamer: ~8 MDa.<br>- Each subunit: Mr ~450 kDa.	- HpH impaired tumor growth while promoting IgG, antibody-producing plasma cells, and tumor-specific CTLs in an animal model of colon carcinoma.<br>- HpH induced NK cells, CTLs, IgM, and type-1 macrophage activation in murine melanoma and colon carcinoma models.<br>- HPH impaired tumor growth and induced apoptosis in colorectal carcinoma cell lines.<br>- HpH stimulated antibody responses against influenza hemagglutinin subunit peptides, tetanus toxoid, and synthetic tumor-associated antigens.	- Carrier protein for the conjugation of small molecules of biotechnological interest.<br>- Immunostimulant and adjuvant in preclinical cancer research.<br>- Immunomodulatory protein used to study the mechanisms of glycan-dependent immune activation and antigen presentation.	- Stoyanova et al. Int Immunopharmacol. 2020..<br>- Yesilyurt et al. FEBS J. 2008.<br>- Stoyanova et al. Mar Drugs. 2024..<br>- Georgieva, et al. Biomedicines. 2020.
PcH	*Pomacea canaliculata*	- Didecameric structure. Stable up to 80°C and from pH ~4.0 to 10.0.<br>- Glycan content: ~2.8% (w/w) including terminal galactose and N-acetylgalactosamine residues, as well as high mannose and complex N-glycans.	- Didecamer: ~8 MDa.<br>- Subunits: Mr ~390 kDa.	- Induced a proinflammatory response in THP-1-derived macrophages in a glycosylation-dependent manner and promoted cell differentiation.<br>- Induced a humoral immune response (IgG) in mice.	An immunomodulatory protein used to study immune activation and antigen presentation.	- Chiumiento et al. PLoS One. 2020.<br>- Chiumiento et al. Front Immunol. 2025.
